# Non-Malignant Granulocyte and Monocyte Disorders: An Update

**DOI:** 10.3389/bjbs.2025.15072

**Published:** 2025-10-10

**Authors:** Sorfina Binti Ahmad Hilmi, Dinesh Kumar Chellappan, Anil Philip Kunnath

**Affiliations:** ^1^ Division of Applied Biomedical Science and Biotechnology, School of Health Sciences, IMU University, Kuala Lumpur, Malaysia; ^2^ Department of Life Sciences, School of Pharmacy, IMU University, Kuala Lumpur, Malaysia; ^3^ Center of Medical and Bio-Allied Health Sciences Research, Ajman University, Ajman, United Arab Emirates

**Keywords:** granulocyte disorders, monocyte disorders, immune response, signalling pathways, genetic testing

## Abstract

Non-malignant disorders of granulocytes and monocytes include a range of conditions characterized by either quantitative issues (such as cytopenias or cytophilias) or qualitative defects in innate immune cells. These disorders encompass neutropenias, monocytopenias, eosinophilic syndromes, and defects in granulocyte maturation. They can result from genetic mutations (including ELANE, HAX1, GATA2, and CSF3R), autoimmune dysregulation, or idiopathic mechanisms. The clinical manifestations of these disorders vary and can include recurrent infections, inflammatory complications, and organ damage. These issues arise from disrupted granulopoiesis, abnormal apoptosis, or dysfunctional chemotaxis. Recent innovations underscore how molecular diagnostics inform both mutation detection and risk stratification in congenital neutropenias. Take ELANE-associated severe congenital neutropenia: such variants not only establish the disorder but also highlight the subsequent hazard of myelodysplastic progression. In contrast, GATA2 deficiency generates isolated monocytopenia, correlating with a broadened window for opportunistic pathogens. Frontline practice now advocates for prompt, integrative assessment using next-generation sequencing alongside quantitative flow cytometry, thereby parsing mild benign states from early clonal hematopoiesis. Management hurdles persist, especially in patients with refractory neutropenia and the calibrated use of immunosuppression in autoimmune etiologies. The COVID-19 pandemic incidentally reiterated the extent of infectious susceptibility within this cohort, prompting the refinement of absolute, personalized prophylactic strategies. This review synthesizes the molecular mechanisms, genetic basis, and therapeutic innovations in non-malignant granulocyte/monocyte disorders, offering a roadmap for personalized management. By bridging mechanistic insights with clinical practice, it addresses unmet needs in diagnostics, risk prediction, and novel biologics, ultimately improving outcomes for these underrecognized yet impactful conditions.

## Introduction

The study of granulocyte and monocyte disorders has seen remarkable progress in recent years, driven by advancements in molecular biology, immunology, and diagnostic technologies. Researchers have gained deeper insights into the underlying mechanisms of these disorders, leading to improved diagnostic accuracy, more targeted therapies, and better patient outcomes [1]. Granulocytes and monocytes are crucial for chronic inflammation, tissue repair, and immune regulation, highlighting the need for ongoing research into non-malignant disorders that impact patient health and treatment outcomes. Clinically, these disorders are associated with various hematologic and inflammatory conditions. Neutrophilia, characterized by elevated neutrophil counts, is linked to the S100A8/A9-NLRP3-IL-1β pathway, which is relevant in myocardial infarction (MI) [2, 3]. Genetic mutations, such as those in AKT1 and JAK1, further influence neutrophilia regulation [4]. In contrast, neutropenia, marked by low neutrophil counts, is often associated with mutations in ELANE and USB1 [5, 6]. Monocytosis, defined by increased monocyte levels, is significant in Chronic Myeloid Leukemia and Chronic Myelomonocytic Leukemia (CML and CMML), with mutations in ASXL1 and NRAS contributing to disease progression [7, 8]. Additionally, congenital disorders like Pseudohypoaldosteronism (PHA), Chediak-Higashi Syndrome (CHS), and Androgen Receptor Deficiency (ARA) exemplify the diverse spectrum of granulocyte and monocyte disorders, underscoring the need for effective diagnostic and therapeutic strategies.

Breakthroughs in technology are driving a new era of precision medicine for non-malignant granulocyte and monocyte disorders. Sophisticated cellular analysis via flow and mass cytometry now allows clinicians to detect subtle functional defects in immune cells, leading to more accurate and earlier diagnoses. This precision is matched by novel therapies, such as JAK1/2 inhibitors, which specifically target the inflammatory signaling responsible for pathological monocytosis in certain patients. Furthermore, next-generation sequencing has been revolutionary in decoding the genetic basis of these diseases, identifying critical mutations that define specific disease subtypes and directly enabling the development of targeted treatments. These converging technologies are transforming patient management from a symptom-based approach to one focused on underlying disease mechanisms. However, there are still significant research gaps, especially when it comes to understanding the molecular mechanisms that govern granulocyte and monocyte differentiation and function. Future research should focus on identifying specific markers for various subsets, clarifying how they interact with other immune cells, and evaluating the effectiveness of new treatment strategies in clinical settings [1, 9]. Filling these gaps is essential for enhancing our understanding of non-malignant granulocyte and monocyte disorders, making this an important update for clinicians and researchers eager to stay informed about the latest developments and future directions in this vital area.

This review provides a thorough update on non-malignant granulocyte and monocyte disorders, highlighting their clinical significance, molecular mechanisms, genetic mutations, new trends, and the need for additional research. We hope to improve knowledge of these disorders and their consequences for patient care by combining existing knowledge and pinpointing areas for further research.

## Neutrophilia

### Genetic Mutations Affecting Neutrophils

Recent studies have revealed important genetic mutations that affect neutrophilia, particularly in genes like AKT1, MAPK14, JAK1, and CD44. AKT1 acts as a key negative regulator of neutrophil recruitment and activation [15]. MAPK14, also known as p38 MAPK, drives cellular responses to stress and inflammation. JAK1 mediates signals from pro-inflammatory cytokines, which directly influence neutrophil growth and development. Additionally, CD44 is a cell surface glycoprotein that is crucial for neutrophil death and is linked to several inflammatory diseases [10]. These genetic findings not only improve our understanding of neutrophilia but also identify potential treatment targets for inflammatory and blood disorders.

### Molecular Mechanisms of Neutrophilia and Therapeutic Implications

Recent research has identified the S100A8/A9-NLRP3-IL-1β axis as a critical driver of neutrophilia and associated tissue damage in pathologies such as cardiovascular disease. The pathway operates through a sequential cascade: the damage-associated molecular patterns (DAMPs) S100A8/A9 activate the NLRP3 inflammasome, culminating in the caspase-1-dependent cleavage and secretion of IL-1β. This cytokine acts centrally to promote both the bone marrow production (granulopoiesis) and peripheral priming of neutrophils, exacerbating inflammation and driving tissue injury [11].

Therapeutically, targeting this axis offers a strategic approach. IL-1 receptor antagonism with agents such as anakinra has shown significant clinical benefit in mitigating mortality in severe hyperinflammatory states, including COVID-19-related acute respiratory distress syndrome (ARDS) [12, 13]. This is supported by a growing body of evidence demonstrating that by blocking the IL-1 receptor, anakinra effectively dampens the overwhelming 'cytokine storm’ that drives systemic inflammation, organ failure, and death in these critically ill patients [14]. Concurrently, novel pharmacologic inhibitors of the NLRP3 inflammasome present an upstream strategy to suppress detrimental inflammation without broadly compromising antimicrobial immunity.

Separately, emerging research highlights the gut microbiome as a critical systemic regulator of inflammation and immunity [15]. Gut microbiota-derived metabolites, such as short-chain fatty acids, have been demonstrated to modulate neutrophil maturation, recruitment, and effector functions. This independent axis of immune regulation unveils a novel therapeutic avenue. Strategies aimed at manipulating the gut microbiome—including probiotics, prebiotics, or fecal microbiota transplantation—are now being investigated for their potential to resolve chronic inflammation across a spectrum of diseases, representing a distinct frontier in immunomodulatory therapy [2, 3].

ORMDL sphingolipid biosynthesis regulator 3 (ORMDL3) is a gene that regulates sphingolipid metabolism, particularly influencing the sphingosine-1-phosphate (S1P) pathway. In respiratory diseases, uncontrolled ORMDL3-dependent sphingosine-1-phosphate (S1P) signalling drives harmful neutrophil movement in severe asthma. This makes S1P receptin modulators, including fingolimod derivatives, promising treatment options [16]. Similarly, in COVID-19, the activation of PFKFB3- a gene that encodes the enzyme 6-phosphofructo-2-kinase/fructose-2,6-biphosphatase 3 in neutrophils by SARS-CoV-2, worsens the unique form of cell death that is characterized by the release of decondensed chromatin and granular contents to the extracellular space called NETosis and cytokine storms. This suggests that metabolic inhibitors may be potential therapies [17].

Cytokine-targeted biologics are gaining significant popularity as precision tools in immunology. This is especially true for therapies targeting the IL-33 pathway, because emerging research shows IL-33 signaling can uniquely suppress neutrophilic inflammation—a key driver of severe, steroid-resistant disease—while simultaneously preserving or even promoting beneficial Type 2 (eosinophil-driven) immune responses, which are critical for parasite clearance and tissue repair [18]. The IL-17/22 pathway also plays a cooperative role in asthma. Additionally, G-CSF’s effects on kidney injury and the inhibition of PI3K by ginsenoside Rg3 in Chronic obstructive pulmonary disease (COPD) show how specific pathway changes can tackle extra-pulmonary neutrophilia [19]. The finding that Signal Transducer and Activator of Transcription 1 (STAT1) suppresses neutrophil recruitment during influenza-fungal co-infections, along with the issue of chemotherapy-induced “neutrophil resonance,” highlights the need for timely interventions.

### Translating Mechanisms Into Therapies

Researchers are leveraging new mechanistic insights to develop targeted clinical applications. A primary strategy involves biomarker-driven patient stratification. For instance, patients most likely to respond to NLRP3 inflammasome inhibitors—which target a key multi-protein complex in innate immunity—could be identified by measuring circulating levels of IL-1β or S100A8/A9 heterodimers. This approach aims to maximize therapeutic effectiveness while minimizing side effects [11, 18].

Another promising avenue in immunomodulation exploits the intricate crosstalk between the host immune system and the microbiome. Pharmaceutical-grade derivatives of specific microbial metabolites, such as polysaccharide A (PSA) from the commensal bacterium *Bacteroides fragilis*, are being actively explored in preclinical and early clinical development. Their significant potential lies in their ability to precisely fine-tune dysregulated neutrophil responses—reducing destructive hyperinflammation in conditions like sepsis or autoimmune diseases—without inducing a state of generalized immunosuppression, thereby leaving critical host defense mechanisms intact [20].

Furthermore, dual-pathway targeting is emerging as a strategy for complex, treatment-resistant inflammatory conditions. For example, combining IL-17 blockade with S1P receptor modulators may yield superior outcomes in severe asthma [21].

Collectively, these advancements are foundational to precision medicine in inflammation, transforming neutrophilia from a passive diagnostic marker into a dynamic, therapeutically modifiable system.

### Novel Testing and Newer Technologies

Recent technological advancements are revolutionizing the study and management of neutrophilia. Cutting-edge techniques such as flow cytometry, combined with 5-ethynyl-2′-deoxyuridine (EdU) incorporation and advanced gating strategies, have enabled precise quantification and characterization of neutrophil populations in myocardial infarction models, shedding light on their recruitment mechanisms [22]. Meanwhile, bioinformatics tools are being leveraged to analyze gene expression data from microarray datasets, facilitating the identification of differentially expressed genes—such as AKT1 and CD44—and their associated pathways in neutrophilia [23]. Additionally, quantitative systems pharmacology (QSP) modeling is providing new insights into neutrophil dynamics during chemotherapy, allowing for optimized treatment regimens that mitigate neutropenia risks [24]. Together, these innovations are not only deepening our mechanistic understanding but also accelerating the development of novel biomarkers and targeted therapies.

### Future Directions

Looking ahead, research into neutrophilia is poised to make significant strides across multiple fronts. The integration of genetic, microbiome, and cytokine profiling will enable true precision medicine approaches, allowing clinicians to tailor neutrophil-modulating therapies to individual patient biology. Metabolic interventions targeting pathways like 6-Phosphofructo-2-kinase/fructose-2,6-bisphosphatase 3 (PFKFB3) inhibition may prove particularly valuable for managing COVID-19-associated neutrophilia and its complications [25]. Simultaneously, engineered microbiome-derived therapeutics, including synthetic microbial metabolites, could offer novel ways to fine-tune neutrophil activity while avoiding broad immunosuppression.

These advances in understanding neutrophilic disorders naturally extend to their clinical counterpart - neutropenia. Just as we’re developing targeted strategies to suppress pathological neutrophilia, parallel innovations are emerging to address deficient neutrophil production and function. The same technologies enabling precise neutrophil modulation - from cytokine profiling to microbiome engineering - may be adapted to boost neutrophil counts in chemotherapy-induced or congenital neutropenias. Furthermore, the real-time monitoring tools being developed for neutropenia could be equally valuable for tracking neutrophil recovery in neutropenic patients. This bidirectional translation of research insights between neutrophilia and neutropenia highlights how advances in one area can inform therapeutic approaches in the other, creating a more comprehensive understanding of neutrophil biology and its clinical management.

### Genetic Mutations in Neutropenia

Recent studies have significantly advanced our understanding of neutropenia by identifying diverse genetic mutations that contribute to its pathogenesis. For instance, two Ethiopian siblings initially misdiagnosed with xeroderma pigmentosum were found to carry a homozygous pathogenic variant in the USB1 gene (c.682C>T; p.Gln228*) and a novel heterozygous mutation in CSF3R (g.160C>T; p.His54Tyr), suggesting an atypical presentation of poikiloderma with neutropenia [26]. In severe congenital neutropenia (SCN), mutations such as ELANE p.Ala79del (c.234_236del) and p.Val197GlufsTer18 (c.589_590insAGGCCGGC) disrupt neutrophil elastase function, while a *de novo* SEC61A1 missense mutation (c.A275G; p.Q92R) further expands the genetic heterogeneity of SCN [27, 28]. Additional ELANE variants (e.g., F218L, R34W) impair enzyme activity, increasing infection susceptibility, whereas mutations in SRPRA and SRP19 highlight the critical role of the signal recognition particle (SRP) complex in neutrophil maturation [29, 30]. Similarly, CSF3R mutations (e.g., c.359G>A, p.G120D) compromise G-CSF receptor signaling, contributing to cyclic neutropenia, and the Wiskott-Aldrich syndrome protein mutations- *WASP-I292T* gain-of-function mutation demonstrates a distinct mechanism in X-linked neutropenia, supported by murine models [31, 32]. These findings underscore the genetic complexity of neutropenia and the need for ongoing refinement of diagnostic screening.

### Molecular Mechanisms and Therapeutic Implications

The disruption of neutrophil homeostasis by Bruton’s tyrosine kinase (BTK) inhibitors in non-malignant contexts involves multiple interconnected pathways, each offering specific therapeutic opportunities. BTK regulates granulopoiesis by modulating PI3K-AKT and MAPK signaling in myeloid progenitors; its inhibition arrests maturation at the myeloblast-promyelocyte stage, though this reversible defect suggests transient PI3K/AKT modulation could restore differentiation [33]. Concurrently, BTK inhibition impairs G-CSF receptor signaling (reduced STAT5 phosphorylation) while enhancing CXCR4-mediated marrow retention, explaining why plerixafor outperforms G-CSF monotherapy in mitigating neutropenia [34]. Off-target effects further exacerbate the condition, as ibrutinib’s suppression of TEC kinase reduces GM-CSF/IL-3 production, though newer agents like acalabrutinib (with lower TEC affinity) exhibit milder myelosuppression [33]. Within the bone marrow niche, BTK maintains progenitor support via NF-κB-dependent stromal cytokine production and β1-integrin anchoring; its inhibition leads to hypocellularity, which experimental cytokine supplementation can reverse, suggesting thrombopoietin agonists (e.g., eltrombopag) might stabilize the niche [34]. Finally, BTK sustains circulating neutrophils by stabilizing Mcl-1 and suppressing caspases via PI3K-AKT/NF-κB. Its inhibition shortens neutrophil lifespan from 6–8 to under 4 h through BAD/caspase-9 activation, implying that AKT inhibitors (e.g., miransertib) could prolong survival [33].

Future therapeutic strategies should prioritize biomarker-guided approaches (e.g., monitoring CXCR4/G-CSF levels and pAKT/pSTAT3 signaling to personalize plerixafor or cytokine therapy), kinase-selective optimization (e.g., BTK/TEC-bispecific inhibitors), niche-targeted interventions (e.g., stromal IL-3/GM-CSF nanoparticles), and apoptosis modulation (e.g., Mcl-1 stabilizers like sabutoclax).

### Novel Testing and Future Directions

Recent innovations in neutropenia diagnostics combine genomic, proteomic, and non-invasive technologies to enhance precision and patient convenience. Whole-genome sequencing (WGS) and mass spectrometry have identified distinct proteomic signatures in SCN, while the ASPEN study’s hierarchical statistical framework (Cochran-Mantel-Haenszel test) has improved the rigor of clinical trial analyses [31, 34, 35]. The Point Check device exemplifies progress in patient-friendly monitoring, using optical imaging to quantify neutrophils transcutaneously, eliminating the need for frequent blood draws [36].

## Eosinophil Disorders

### Genetic Mutations in Eosinophilia

Recent advances have identified several pathogenic mutations contributing to eosinophilia through distinct molecular mechanisms. The JAK2_ex12InDel mutation causes a 4-amino acid deletion with variable 1-amino acid insertion in the JH2 domain, disrupting autoinhibition and leading to constitutive STAT5/ERK activation through IL-3R/IL-5R independent of ligand binding [37]. This results in uncontrolled eosinophil production and survival, often accompanied by erythrocytosis, suggesting phenotypic overlap with polycythemia vera [36]. Another significant finding is the JAK1 R629_S632delinsA mutation in the pseudokinase domain, which confers growth factor independence through persistent JAK-STAT activation and demonstrates responsiveness to ruxolitinib in clonal eosinophilia [38].

The TrkA F921 mutation in the ATP-binding pocket alters eosinophil trafficking by enhancing spreading while inhibiting eotaxin-1-mediated migration, presenting a novel therapeutic target for allergic asthma [39]. In primary immunodeficiencies, the STAT3 c.1293A>T missense mutation disrupts immune regulation, leading to AD-HIES characterized by elevated IgE and eosinophilia [40]. IL5RA variants in eosinophilic esophagitis (EoE) enhance IL-5 receptor signaling, driving tissue inflammation through exaggerated eosinophilic responses [41–43]. These findings collectively reveal the genetic complexity underlying eosinophilic disorders.

### Molecular Mechanisms of Eosinophilia

The pathogenesis involves dysregulated signaling pathways that promote eosinophil differentiation, survival, and tissue infiltration. Constitutive JAK-STAT activation [37, 38] bypasses normal cytokine regulation, while TrkA F921 alters chemotactic responses to eotaxin-1 [39]. In AD-HIES, defective STAT3 signaling [40] promotes Th2 polarization and IL-5-driven eosinophilopoiesis, whereas IL5RA mutations [41–43] hypersensitize eosinophils to IL-5 stimulation. These mechanisms demonstrate how diverse genetic lesions converge on common inflammatory pathways.

### Therapeutic Implications

Recent molecular insights have revolutionized therapeutic approaches for eosinophilic disorders through targeted interventions. JAK inhibitors such as ruxolitinib demonstrate significant efficacy in JAK1/2-mutant eosinophilia by suppressing constitutive signaling pathway activation [38]. For allergic inflammation, emerging TrkA-targeted therapies show promise in normalizing pathological eosinophil migration patterns while preserving physiological immune functions [44]. Monoclonal antibodies against the IL-5 pathway, particularly mepolizumab, achieve dramatic eosinophil reduction in IL5RA-associated eosinophilic esophagitis by interrupting this critical cytokine-receptor axis [41–43]. In the context of immune dysregulation, novel STAT3 modulators offer potential for correcting the underlying Th2 skewing characteristic of AD-HIES while maintaining protective immunity [40]. These precision medicine approaches collectively highlight the essential role of comprehensive genetic and molecular profiling in guiding therapeutic decision-making for eosinophilic disorders, moving beyond empirical treatment toward mechanism-based interventions tailored to each patient’s specific pathophysiology.

### Diagnostic Advancements

Modern genomic technologies have revolutionized eosinophilia evaluation, with whole exome sequencing (WES) uncovering pathogenic STAT6 mutations in pediatric allergic cases [45, 46] and next-generation sequencing (NGS) identifying disease-associated STAT1 variants in familial eosinophilic esophagitis (EoE) [47]. The integration of fluorescence *in situ* hybridization (FISH) with NGS has proven particularly valuable for characterizing PDGFRB rearrangements and detecting somatic mutations in genes like TET2 and DNMT3A in myeloid-associated eosinophilia [48, 49]. Complementary functional approaches, including immunofluorescence microscopy and Western blot analysis, have further elucidated the protein-level consequences of mutations such as TrkA F921 on eosinophil behavior and signaling pathways [39]. Together, these advanced techniques provide a multidimensional assessment of eosinophilic disorders, enabling clinicians to establish precise molecular diagnoses and tailor therapeutic strategies to individual patients’ genetic profiles.

### Future Directions

While eosinophilia mechanisms are increasingly defined, eosinopenia pathogenesis remains less understood. Preliminary evidence suggests IL-5 signaling defects, glucocorticoid excess, or CEP112 mutations may impair eosinopoiesis. Emerging single-cell technologies may reveal shared regulatory mechanisms between these opposing conditions, potentially identifying novel therapeutic targets for eosinophil homeostasis disorders.

### Genetic Factors in Eosinopenia

Eosinopenia (eosinophil counts <100/μL) has emerged as a significant clinical biomarker, particularly in infectious and respiratory diseases. In COVID-19, persistent eosinopenia shows strong prognostic value, present in 86% of non-survivors compared to only 50% of survivors [50], with evidence suggesting type 1 interferon-mediated suppression of eosinopoiesis may contribute to this association [51]. The biomarker demonstrates particular clinical utility in acute exacerbations of COPD (AECOPD), where its combination with lymphocytopenia shows 100% sensitivity for predicting mortality [52]. Furthermore, eosinopenia proves valuable for infection detection in patients receiving IL-6 pathway antagonists like tocilizumab, where traditional inflammatory markers may be suppressed [53]. These findings collectively establish eosinopenia as an accessible and clinically relevant indicator of disease severity across multiple pathological contexts.

### Molecular Mechanisms of Eosinopenia

The pathophysiology of eosinopenia involves complex regulatory mechanisms, best characterized in glucocorticoid-induced cases. Research using rhesus macaque models - which closely mirror human responses unlike rodent models - reveals that glucocorticoids rapidly upregulate CXCR4 on eosinophils within 1–2 h, facilitating their migration to CXCL12-rich bone marrow niches [54]. This mechanism was conclusively demonstrated through innovative ^89^Zr-oxine PET imaging techniques, with CXCR4 blockade by plerixafor completely abolishing the glucocorticoid-induced eosinopenic effect [55]. The specificity of this response highlights both the importance of model selection in eosinophil research and the potential for targeted therapeutic interventions modulating this trafficking pathway.

### Therapeutic Implications and Diagnostic Approaches

The clinical management of eosinopenia-related conditions benefits from these mechanistic insights, particularly in monitoring infections in immunocompromised patients, where eosinophil thresholds below 0.05 G/L serve as reliable indicators [53]. Diagnostic capabilities have been significantly enhanced by technological advances, including ^89^Zr-oxine PET imaging for real-time eosinophil tracking and immunomagnetic isolation techniques that permit high-purity eosinophil studies [54]. These developments not only improve our understanding of eosinophil dynamics but also open possibilities for targeted therapies, such as CXCR4 modulation to manage glucocorticoid side effects while maintaining therapeutic efficacy [55].

As our understanding of eosinophil regulation advances, parallel investigations into basophil disorders - encompassing both basophilia and basopenia - become increasingly pertinent. The technological and mechanistic insights gained from eosinopenia research, particularly regarding granulocyte trafficking and biomarker applications, may prove equally valuable in elucidating basophil pathophysiology in allergic, infectious, and inflammatory conditions.

## Basophil Disorders

### Basophilia

#### Genetic Factors in Non-Malignant Basophilia

While non-malignant basophilia lacks the driver mutations characteristic of myeloid neoplasms, recent studies have identified key genetic and cytokine-mediated mechanisms underlying reactive basophil expansion. Germline variants in STAT5B have been associated with enhanced basophil production in rare genetic disorders, demonstrating how inherited alterations can influence basophil homeostasis without malignant transformation [54]. More commonly, dysregulation of cytokine signaling pathways - particularly involving IL-3 and thymic stromal lymphopoietin (TSLP) - drives basophil differentiation and survival in allergic and inflammatory conditions [56]. The IgE-mediated FcεRI activation pathway has also been shown to promote basophil hyperplasia in hypersensitivity reactions, independent of clonal mutations [57]. Current research focuses on identifying specific cytokine profiles (e.g., IL-4, IL-13) and biomarkers like CD203c that distinguish reactive basophilia from neoplastic variants while providing prognostic information in conditions such as chronic urticaria and atopic dermatitis [58].

#### Molecular Mechanisms of Reactive Basophilia

The pathophysiology of non-malignant basophilia involves complex interactions between immune signaling pathways and tissue microenvironment factors. In parasitic infections, IL-3 and TSLP play pivotal roles in basophil recruitment and activation, with Notch signaling further enhancing basophil responses during type 2 inflammation [59]. TSLP-elicited pathways appear particularly important in driving both inflammatory and tissue repair functions of basophils, creating a dual role in maintaining immune homeostasis. The surface marker CD203c has emerged as a reliable indicator of basophil activation status, with expression levels correlating with disease severity in allergic conditions [58]. Unlike in malignant basophilia, these mechanisms operate through physiological cytokine networks rather than autonomous clonal proliferation, preserving the beneficial roles of basophils in parasite defense and tissue repair while contributing to pathological inflammation in allergic diseases.

#### Therapeutic Implications and Targeted Approaches

Advances in understanding these molecular mechanisms have led to several targeted therapeutic strategies for managing non-malignant basophilia. Anti-IgE therapy with omalizumab has demonstrated efficacy in severe allergic conditions by disrupting FcεRI-mediated basophil activation while maintaining protective immune functions [59]. Emerging approaches focus on modulating specific cytokine signals, including IL-3 and TSLP inhibition, to reduce basophil-mediated inflammation in chronic urticaria and atopic dermatitis. Kinase inhibitors targeting SYK and BTK show promise in dampening FcεRI signaling pathways without completely abolishing basophil activity [58]. These targeted interventions aim to strike a balance between controlling pathological inflammation and preserving the beneficial roles of basophils in host defense and tissue repair, representing a significant advance over non-specific immunosuppressive therapies. Current research continues to explore biomarkers like basogranulin for monitoring disease activity and treatment response in these non-malignant basophilic disorders.

#### Genetic Factors in Non-Malignant Basopenia

While the genetic basis of non-malignant basopenia remains less characterized than other cytopenias, emerging research has identified key immunological patterns. In chronic spontaneous urticaria (CSU), an autoimmune component is suggested by low baseline IgE levels and reduced FcεRI receptor expression, which correlate with basophil depletion [60]. Mixed connective tissue disease (MCTD) presents a distinct profile, where approximately 80% of patients demonstrate autoreactive IgE against U1-snRNP alongside peripheral basopenia, indicating a specific autoimmune etiology [61]. Notably, mRNA vaccinations may exacerbate basopenia in CSU through autoimmune activation, though remaining basophils maintain functional activity [62]. These findings highlight the complex interplay between genetic predisposition and autoimmune mechanisms in basopenia development.

#### Molecular Mechanisms of Basopenia

The pathophysiology of non-malignant basopenia involves multiple interconnected pathways. In CSU, autoantibodies against FcεRI promote basophil activation and subsequent depletion through intravascular mechanisms while simultaneously stimulating histamine release [63]. Prostaglandin D2 (PGD2) signaling plays a dual role, both attracting basophils to affected tissues and contributing to their peripheral depletion [64]. MCTD demonstrates unique basophil trafficking patterns, with CCR3 overexpression driving migration to lung tissue despite systemic basopenia [65]. These mechanisms collectively explain the paradoxical findings of both basophil depletion and hyperactivity observed in autoimmune conditions, with severity often correlating with resistance to conventional antihistamine therapy [64].

#### Therapeutic Implications and Diagnostic Approaches

Current management of non-malignant basopenia emphasizes targeted modulation of autoimmune pathways, with anti-IgE therapy representing a cornerstone approach. Omalizumab demonstrates particular efficacy in chronic spontaneous urticaria (CSU) by normalizing FcεRI expression and restoring basophil homeostasis, while simultaneously reducing symptom severity [62, 64]. For patients with complex autoimmune presentations, personalized regimens including adjusted antihistamine dosing or cyclosporine may provide additional clinical benefit, particularly in cases showing resistance to first-line therapies [7]. Emerging diagnostic tools are revolutionizing patient stratification, where flow cytometry analysis of basophil activation patterns and autologous serum skin testing (ASST) enable precise identification of pathogenic autoantibodies [7, 8, 64]. These advancements facilitate treatment customization, notably in distinguishing omalizumab responders from non-responders through characterization of basophil surface markers and IgE receptor profiles [7, 64]. The integration of functional basophil assessment with therapeutic monitoring underscores the cell’s dual role as both a pathogenic mediator and treatment response biomarker in autoimmune-associated basopenia.

The immunological insights gained from studying basopenia - particularly regarding autoimmune-mediated cytopenias and trafficking abnormalities - provide a valuable framework for investigating parallel mechanisms in monocyte disorders. Similar approaches combining functional assays with targeted therapies may prove equally informative in understanding conditions characterized by monocytopenia or dysfunctional monocyte activation.

### Non-Malignant Monocyte Disorders

#### Genetic Mutations in Non-Malignant Monocytosis

Several genetic mutations contribute to reactive monocytosis in non-malignant conditions. Activating mutations in CSF1R enhance sensitivity to colony-stimulating factor 1 (CSF1), driving excessive monocyte production and tissue infiltration that sustains chronic inflammation [65, 66]. Germline mutations in SAMD9/SAMD9L disrupt hematopoietic regulation, with compensatory monocytosis often preceding bone marrow failure manifestations [67]. Gain-of-function STAT3 mutations promote monocyte overproduction through hyperactive IL-6/GM-CSF signaling, creating a pro-inflammatory loop in autoinflammatory syndromes [68]. These genetic alterations share a common pathway of disrupting normal monocyte homeostasis without malignant transformation.

#### Molecular Mechanisms of Reactive Monocytosis

The pathophysiology involves cytokine-driven myelopoiesis and altered monocyte trafficking. CSF1R hyperactivation expands the CD14^+^ classical monocyte pool, which exhibits enhanced CCR2-mediated migration to inflamed tissues [66]. In SAMD9/SAMD9L disorders, stress-induced hematopoietic reprogramming shifts differentiation toward monocytic lineages as a compensatory mechanism [67]. STAT3-mediated monocytosis involves JAK-STAT hyperactivation that simultaneously increases bone marrow output and promotes inflammatory polarization through IL-23/IL-17 axis stimulation [68]. These mechanisms collectively demonstrate how dysregulated cytokine signaling and transcriptional control can drive pathogenic monocyte expansion in non-neoplastic contexts.

#### Therapeutic Implications

Current management of non-malignant monocytosis employs targeted strategies addressing specific molecular pathways, beginning with CSF1R inhibition using tyrosine kinase inhibitors such as pexidartinib to curb pathological monocyte infiltration in inflammatory disorders driven by CSF1R hyperactivation [69]. For STAT3-mediated conditions, JAK-STAT modulation with ruxolitinib has proven effective in controlling both monocyte overproduction and associated inflammatory cascades, particularly in autoinflammatory syndromes characterized by IL-23/IL-17 axis dysregulation [70]. Cytokine blockade remains a cornerstone approach, where IL-1 inhibitors (anakinra) and IL-6 receptor antagonists (tocilizumab) attenuate monocyte-mediated tissue damage by interrupting key inflammatory signaling loops [69]. Supportive care measures, including tailored antimicrobial prophylaxis, remain essential to prevent opportunistic infections during immunosuppressive therapies, particularly in patients with concurrent immune dysfunction [71]. Emerging precision medicine approaches now explore CRISPR-Cas9 gene editing to correct SAMD9/SAMD9L mutations in preclinical models, while advanced monitoring techniques like single-cell RNA sequencing enable real-time treatment optimization by tracking monocyte subset responses to therapy [72, 73]. These stratified approaches demonstrate how a mechanistic understanding of monocytosis pathogenesis translates to targeted clinical interventions.

While monocytosis reflects excessive myeloid activation, the opposite condition – monocytopenia – arises from distinct genetic defects impairing monopoiesis. Disorders like GATA2 and IRF8 deficiencies demonstrate how developmental defects in progenitor cells lead to clinically consequential monocyte deficiency, creating a spectrum of dysregulation that will be explored next.

#### Genetic Mutations in Non-Malignant Monocytopenia

Monocytopenia reveals critical insights into immune dysfunction through key genetic mutations. GATA2 deficiency, caused by mutations like c.177C>A (p.Y59) and c.610dup (p.R204Pfs78), disrupts hematopoietic stem cell maintenance, leading to progressive monocyte loss and severe immunodeficiency [74–76]. Warts hypogammaglobulinemia immunodeficiency myelokathexis syndrome (WHIM syndrome) demonstrates how CXCR4 gain-of-function mutations (e.g., p.V320FS342X) impair leukocyte trafficking through abnormal bone marrow retention [77].

IRF8 mutations (c.331C>T) truncate this essential transcription factor, blocking monocyte differentiation and predisposing to mycobacterial infections [78]. Other significant variants include RAP1B c.35G>A (p.G12E) altering integrin signaling [79] and GATA2 c.1009C>T (p.Arg337*) associated with hyperinflammatory syndromes [80]. These findings underscore the necessity of comprehensive genetic screening in unexplained cytopenias.

#### Molecular Mechanisms of Monocytopenia

The pathophysiology involves multilayered disruptions to monopoiesis. GATA2 deficiency creates a transcriptional void in myeloid progenitors, impairing their commitment to monocytic lineages while simultaneously reducing dendritic cell output [76, 81]. Hypoxia, as seen in ARDS, suppresses type I interferon-dependent survival signals, skewing monocyte polarization toward immunosuppressive phenotypes [81]. CXCR4 hyperactivation in WHIM syndrome traps neutrophils and monocytes in bone marrow through excessive SDF-1 binding [72]. RAP1B mutations exemplify signaling paradoxes—enhanced integrin-mediated adhesion coexists with defective hematopoiesis [82]. These mechanisms converge on mTOR/NF-κB pathways, suggesting shared therapeutic targets despite diverse etiologies.

#### Therapeutic Strategies

Management requires tailored approaches addressing specific defects. CSF-1 supplementation restores macrophage function in hypoxic conditions like ARDS by bypassing impaired differentiation [79]. Plerixafor mobilizes sequestered leukocytes in WHIM syndrome by antagonizing pathogenic CXCR4 signaling [81, 83]. For genetic etiologies, reduced-toxicity allogeneic hematopoietic cell transplantation (allo-HCT) can reconstitute normal hematopoiesis in GATA2 deficiency [75, 84], while combined immunosuppression and antiviral therapy controls hyperinflammation in GATA2-associated HLH [76]. Emerging immunomodulators targeting mTOR/NF-κB pathways may offer broader applicability across monocytopenia subtypes.

#### Diagnostic Advancements

Modern diagnostic technologies have revolutionized the evaluation of monocytopenia through complementary approaches. Multiparametric flow cytometry now enables precise quantification of monocyte subsets and their functional states, proving particularly valuable for guiding immunomodulatory therapy in ARDS and COVID-19 patients where monocyte dynamics predict clinical outcomes [82, 85]. The advent of high-throughput genomic sequencing platforms like Illumina HiSeq X10 has transformed genetic diagnosis, allowing identification of causative mutations such as IRF8 c.331C>T with 99.9% accuracy while simultaneously screening for associated immune deficiencies [77]. Functional assays have evolved beyond basic phenotyping to assess specific pathway abnormalities, including CXCR4 activation status and mTOR signaling flux, enabling truly personalized treatment selection based on a patient’s molecular profile. These technological advances collectively provide a diagnostic framework that bridges genetic etiology with functional consequences in monocytopenia.

## Conclusion

This comprehensive analysis reveals fundamental parallels across non-malignant granulocyte and monocyte disorders, where genetic lesions in transcription factors (GATA2, IRF8), cytokine receptors (CSF1R, CXCR4), and signaling molecules (STAT3, RAP1B) disrupt myeloid homeostasis through converging pathways. The shared clinical themes – infection susceptibility, inflammatory dysregulation, and hematopoietic instability – underscore the need for integrated diagnostic approaches combining advanced genomics (WES/WGS), functional immunophenotyping, and pathway activity mapping. Emerging therapeutic strategies similarly transcend individual disorders, with JAK/STAT inhibition, cytokine modulation, and targeted gene correction offering potential across eosinophil, basophil, neutrophil, and monocyte pathologies. Future progress hinges on three key areas: (1) development of unified classification systems incorporating molecular and functional data, (2) clinical trials of pathway-specific therapies (mTOR inhibitors, CXCR4 antagonists) in genetically stratified cohorts, and (3) advancement of CRISPR-based gene editing for curative approaches. By recognizing these disorders as interconnected manifestations of myeloid dysregulation rather than isolated entities, we can accelerate the translation of basic discoveries into precision medicine solutions that improve outcomes for all patients with non-malignant granulocyte and monocyte disorders.
